# Echocardiographic Parameters for Risk Prediction in Borderline Right Ventricle: Review with Special Emphasis on Pulmonary Atresia with Intact Ventricular Septum and Critical Pulmonary Stenosis

**DOI:** 10.3390/jcm12144599

**Published:** 2023-07-10

**Authors:** Massimiliano Cantinotti, Colin Joseph McMahon, Pietro Marchese, Martin Köstenberger, Marco Scalese, Eliana Franchi, Giuseppe Santoro, Nadia Assanta, Xander Jacquemyn, Shelby Kutty, Raffaele Giordano

**Affiliations:** 1 Fondazione G. Monasterio CNR-Regione Toscana, 56124 Pisa, Italy; cantinotti@ftgm.it (M.C.); pmarchese@ftgm.it (P.M.); eliana.franchi@ftgm.it (E.F.); giuseppe.santoro@ftgm.it (G.S.); assanta@ftgm.it (N.A.); 2 Institute of Clinical Physiology, 56124 Pisa, Italy; 3 Department of Pediatric Cardiology, Childrens Health Ireland, D12 N512 Dublin, Ireland; colin.mcmahon@olchc.ie; 4 School of Medicine, University College Dublin, D04 V1W8 Dublin, Ireland; 5 Istituto di Scienze Della Vita (ISV), Scuola Superiore Sant’Anna, 56127 Pisa, Italy; scalese@ifc.cnr.it; 6 Department of Pediatrics, Division of Pediatric Cardiology, Medical University Graz, 8036 Graz, Austria; martin.koestenberger@medunigraz.at; 7 Helen B. Taussig Heart Center, Department of Pediatrics, Johns Hopkins Hospital, Baltimore, MD 21205, USA; skutty1@jhmi.edu (X.J.); shelby.kutty@gmail.com (S.K.); 8 Adult and Pediatric Cardiac Surgery, Department Advanced Biomedical Sciences, University of Naples “Federico II”, 80131 Naples, Italy

**Keywords:** echocardiography, borderline right ventricles, scores, pediatrics

## Abstract

The aim of the present review is to highlight the strengths and limitations of echocardiographic parameters and scores employed to predict favorable outcome in complex congenital heart diseases (CHDs) with borderline right ventricle (RV), with a focus on pulmonary atresia with intact ventricular septum and critical pulmonary stenosis (PAIVS/CPS). A systematic search in the National Library of Medicine using Medical Subject Headings and free-text terms including echocardiography, CHD, and scores, was performed. The search was refined by adding keywords “PAIVS/CPS”, Ebstein’s anomaly, and unbalanced atrioventricular septal defect with left dominance. A total of 22 studies were selected for final analysis; 12 of them were focused on parameters to predict biventricular repair (BVR)/pulmonary blood flow augmentation in PAIVS/CPS. All of these studies presented numerical (the limited sample size) and methodological limitations (retrospective design, poor definition of inclusion/exclusion criteria, variability in the definition of outcomes, differences in adopted surgical and interventional strategies). There was heterogeneity in the echocardiographic parameters employed and cut-off values proposed, with difficultly in establishing which one should be recommended. Easy scores such as TV/MV (tricuspid/mitral valve) and RV/LV (right/left ventricle) ratios were proven to have a good prognostic accuracy; however, the data were very limited (only two studies with <40 subjects). In larger studies, RV end-diastolic area and a higher degree of tricuspid regurgitation were also proven as accurate predictors of successful BVR. These measures, however, may be either operator and/or load/pressure dependent. TV Z-scores have been proposed by several authors, but old and heterogenous nomograms sources have been employed, thus producing discordant results. In summary, we provide a review of the currently available echocardiographic parameters for risk prediction in CHDs with a diminutive RV that may serve as a guide for use in clinical practice.

## 1. Background

Borderline right ventricle (RV) encompasses a wide spectrum of complex congenital heart diseases (CHDs) with neonatal presentation including pulmonary atresia with intact ventricular septum (IVS), critical pulmonary stenosis (CPS), [1,2,3,4,5,6,7,8,9,10,11,12,13] severe Ebstein’s anomaly [14,15,16,17,18,19,20,21,22], and unbalanced atrioventricular septal defect (uAVSD) with left dominance [23,24,25,26]. Echocardiography is the foremost and often only imaging modality employed in the diagnosis and estimation of disease severity, which influences surgical/interventional planning in these complex defects. Despite this, echocardiographic parameters and scores for disease severity and risk prediction in complex CHDs with borderline RV are lacking and heterogeneous in nature. Several studies have tried to evaluate which echocardiographic measures were able to predict favorable outcomes, including successful biventricular repair (BVR) and the need for pulmonary blood flow stabilization/re-intervention, in neonatal CHDs with borderline RV [1,2,3,4,5,6,7,8,9,10,11,12,13,14,15,16,17,18,19,20,21,22,23,24,25,26]. Most of the literature is focused on pulmonary atresia IVS and CPS [1,2,3,4,5,6,7,8,9,10,11,12,13], whereas very few studies are available for Ebstein’s anomaly [18,21] and uAVSD with left dominance [25,26]. Consequently, our aim was to review echocardiographic parameters predictive of a favorable outcome in borderline RV, with special attention on those parameters most predictive for successful BVR. We also investigated which variables predicted the need for duct stenting/shunt palliation after percutaneous balloon pulmonary valvuloplasty in PAIVS and CPS.

## 2. Methods

In June 2022, we performed a systematic search in the National Library of Medicine for Medical Subject Headings and free-text terms including “echocardiography,” “congenital heart disease”, and “scores”. The search was refined by adding keywords for “pulmonary atresia intact ventricular septum” and “critical pulmonary stenosis,” “ Ebtsein’s anomaly”, and “uAVSD with left dominance”. The titles and abstracts of articles identified were evaluated and excluded if: [1] the reports were written in languages other than English (1 study) and [2] studies did not report echocardiographic scores (25 studies).

## 3. Results

**Figure 1:**
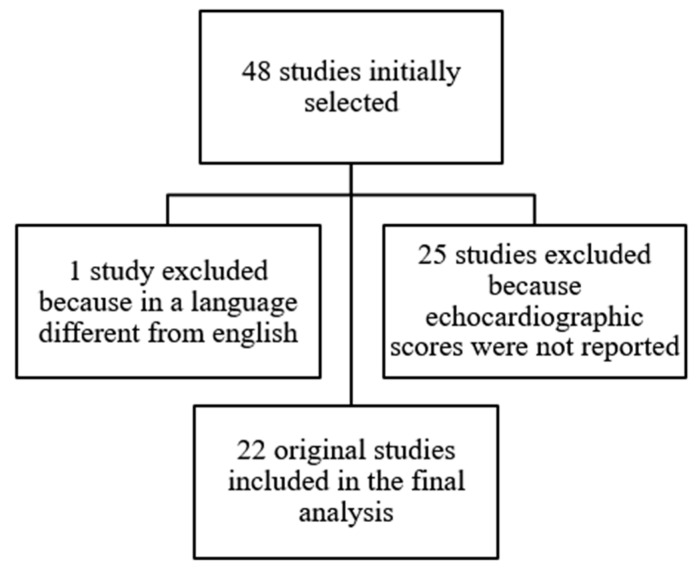
Study selection.

From 48 studies initially selected, 22 original studies [1,2,3,4,5,6,7,8,9,10,11,12,13,16,17,18,19,20,21,23,25,26] met the criteria established and were selected for analysis (Figure 1).

### 3.1. Pulmonary Atresia with Intact Ventricular Septum and Critical Pulmonary Stenosis

#### 3.1.1. General Methodological Limitations

Echocardiographic parameters able to determine favorable immediate [2,3,4,6,10] and long-term outcome in neonates with PAIVS and CPS were analyzed [1,5,11]. All studies were retrospective in nature, and most comprised of single-center designs with limited exceptions [1,2,12]. Sample size was limited (varying from 22 to 36 subjects) [4,5,7,9,10] or relatively limited (varying from 53 to 99 subjects) [2,6,8,11].

Inclusion criteria were different in most studies, including only those children undergoing percutaneous balloon pulmonary valvuloplasty as an initial procedure [3,4,5,6,8,9,10]. Most of the studies were focused only on PA IVS undergoing percutaneous balloon pulmonary valvuloplasty (PBPV) [1,3,5,8,9,16], whereas some had more exhaustive inclusion criteria such as children treated with both PBPV and surgically with a Blalock–Thomas–Taussig shunt (BTT) [11] or those only [13] treated surgically, either with BTT or closed trans-ventricular valvulotomy [13]. Few studies also included CPS [3,4], whereas one was limited to children with CPS [6].

Endpoints were also different between studies, comprising of the need for PDA stenting or BTT [3,4,6,8] reintervention [2,6,10] and good biventricular outcome [1,5,11,13]. Whereas most of the authors evaluated only neonates [1,2,4,6,8,9,10,11], others additionally included older children [5,8,13] up to 8 years of age.

Exclusion criteria were often poorly defined, and when they were specified [2,8,11,12,13,14], they also varied between different authors. They consisted of decompression after the neonatal period [1,2], previous BTT [5], RV-dependent coronary circulation [3,5], unipartite RV [5] or diminutive RV [3,10], and muscular infundibular atresia [3,9]. Neonates with Ebstein’s anomaly were excluded from four cohorts [1,2,5,10], whereas this was not specifically described in other studies [6,8,13]. The echocardiographic parameters evaluated also varied among different authors, as detailed in Table 1 and Supplementary Table S1.

#### 3.1.2. Echocardiographic Parameters Evaluated

##### The Tricuspid Valve

- Tricuspid valve Z-score

Many authors used TV Z-scores as an indicator for the need of PDA stenting or BTT [3,4,6], re-intervention [11], or BVR success [13]. The specific Z-scores employed, however, differed among various studies. Often the source of Z-score [27,28,29,30,31,32] was not provided [3,6,8,10], whereas a few authors used very old (e.g., Rowllat) [13,17] or old formulas (e.g., Daubeney) [4,5,28]. Only a few authors [1,2,4] used recently provided Z-scores (e.g., Pettersen) [31]. The use of different nomograms (especially older ones) may generate discordant ranges of normality, thus generating confusion in disease severity estimation. It is well known that nomograms employed for years have important limitations, providing a range of normality widely different from those obtained with the more recently proposed Z-scores [27,31,32]. To provide a practical example: in a baby of 3 kg and 50 cm with a given TV annulus of 10 mm, the Z-score varied from −3.8 using older Z-scores (Daubeney) [15], to −1.59 (Cantinotti) [27], to −1.24 (Pettersen) [31], and up to −0.55 (Lopez) [32] using more recent nomograms. Thus, it is not surprising that the range/cut-off values for the TV Z-score that indicated a higher risk for PA IVS/CPS varied greatly among different studies. For instance, in terms of the need for PDA stenting or surgical shunt, the critical TV Z-scores cut-off values varied from ≤0.7 to −2.12 [3,4,5,8,11] according to the different authors. Those authors who employed older Z-score sources [15] tended to overestimate the degree of hypoplasia [12], suggesting that even children with very low TV scores (up to −5) had the possibility for successful BVR. Authors who employed more recent nomograms (e.g., Pettersen) [31] demonstrated that even mild TV hypoplasia (a cut-off value TV Z-score ≤ 0.74 having a specificity of 90% and a sensitivity of 77.8%) [4] was predictive of the need for PDA stenting/shunt in 36 neonates and infants with PAIVS/CPS who underwent CPBP. Despite these findings, TV hypoplasia is considered a negative indicator [4,11]; Petit and colleagues [2] demonstrated that in 99 patients with PAIVS, a larger TV annulus was associated with a higher risk of reintervention for restenosis of the right ventricular outflow tract (RVOT) (hazard ratio, 1.20 per 1 mm increase; *p* = 0.071).

- TV/MV annular ratio

**Figure 2:**
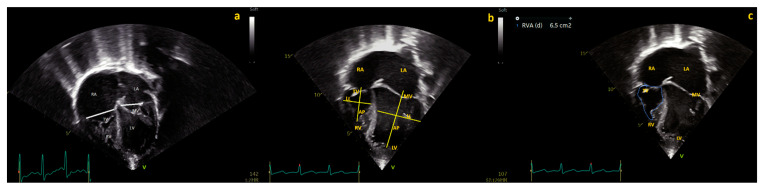
Four chamber view of a PAIVS with diminutive, hypertrophied RV. (**a**) Mitral valve (MV) and tricuspid valve (TV) diameters. (**b**) Base–apex and laterolateral diameters of the right (RV) and left ventricles (LV), (**c**) right ventricle end-diastolic (RVED) area.

The use of the TV/MV annular ratio may overcome the issues related to the use of different Z-scores. The TV/MV annular ratio is an easy and very reproducible measurement, independent of external formulas, that has been employed by several authors [3,4,5,13] (Figure 2). Similar to TV Z-scores, the cut-offs indicated by different authors varied widely. A few studies demonstrated that children with mild [23] (e.g., TV/MV ratio > 0.79) or even moderate [13] TV hypoplasia (e.g., TV/MV ratio ≥ 0.5) could achieve a successful BVR. Paradoxically, other studies reported that even a very mild (e.g., TV/MV ratio ≤ 0.9) [4] or mild ratio (e.g., TV/MV ratio < 0.78) [3] was predictive of the need for PDA stenting or BT shunt to stabilize the pulmonary blood flow [3,4].

- Tricuspid regurgitation

The degree of tricuspid regurgitation (TR) is an important indicator for successful BVR [1,2]. Maskatia et al. [1] found that in 81 neonates with PAIVS treated with PBPV, a greater than moderate TR was associated with RV growth. Similarly, mild or less than mild [2] TR was associated with the need for re-intervention and failing BVR in 99 neonates with PAIVS. Other authors [3] have indicated that the presence of moderate/severe TR was a risk factor for duct dependency of the pulmonary circulation. It is important to remember that the criteria for grading TR in the pediatric age by echocardiography, especially at younger neonatal ages, are not completely defined [33,34]. Consequently, different criteria to grade TR have been adopted by several authors. Maskatia et al. [1], graded the TR as none/mild or moderate/severe according to vena contracta, jet area, deceleration time, and flow reversal in pulmonary arteries; however, the cut-off values for inclusion in different categories are described to be not indicated [1]. Other studies [2,3] refer to respective guidelines, however, pediatric guidelines for the quantitative echocardiographic evaluation of TR are not currently available [33,34] and adult guidelines are often extrapolated to children without any validation [35].

##### Right Ventricle Size

Evaluation of RV size is difficult at echocardiography and may be even more difficult in hypoplastic, hypertrophied bipartite RV, such as those in neonates with PAIVS.

- RV diameters

An easy way to measure RV dimensions is to calculate the lateral diameter and base–apex length in the four-chamber view (Figure 2). The RV/LV lateral diameters ratio has been tested for prediction of BVR by Drighill and colleagues [10], who retrospectively evaluated 26 patients with PAIVS who underwent PBPV at a single institution (13 with successful BVR, 13 with unsuccessful BVR). The authors [10] demonstrated that an RV/LV diameter > 0.76 predicted a 92.3% success rate for BVR. When the RV/LV diameter was ≤0.76 and RV/LV length was >0.70, the success rate for BVR dropped to 75%. When both the RV/LV diameter was ≤0.70 and the RV/LV length was ≤0.76, there was a 100% probability of failure for BVR. Additionally, the RV length determined by the four-chamber view [2] was one of the major determinants for any re-intervention after decompression in 99 children with PAIVS undergoing PBPV (hazard ratio-HR-, 0.94; for 0.01 mm, 95% CI, 0.89–0.99; *p* = 0.027) [2].

- Right ventricle end-diastolic (RVED) area

Another technique to measure RV size by echocardiography is to trace the end-diastolic area in the four-chamber view. However, the tracing of endocardial borders may be quite difficult in very hypertrophied, trabeculated RV such as those of neonates with PAIVS/CPS (Figure 2). RV areas and volumes in the neonatal age are greatly influenced by load and pressure conditions and may vary greatly from one moment to the other. Therefore, the cut-off for the RVED area proposed by different authors differed widely from 6 cm^2^/m^2^ for BVR [1] to 1.35 cm^2^ for ductal dependency after PBPV [3]. Maskatia et al. [1] investigated 81 neonates who underwent PBPV for PAIVS and demonstrated that an RVED area at baseline ≥ 6 cm^2^/m^2^ had a sensitivity of 93% and a specificity of 80% in predicting BVR. Petit and colleagues [2] showed that the RVED area was lower in neonates with PAIVS who underwent univentricular palliation (*n* = 17) compared with those who underwent biventricular repair (*n* = 82) (mean 1.0 cm^2^, range 0.9 to 1.2 cm^2^ in UVP versus 2.1 cm^2^, range 1.6 to 2.6 cm^2^ in BVR, *p* < 0.0001). The RVED area had an odds ratio of 0.81 per 0.1 cm^2^ increase (95% CI, 0.72–0.91; *p* < 0.001) for the prediction of BVR, however, cut-off values were not proposed (2). Lastly, Giordano et al. indicated a value for the RVED area of 1.35 cm^2^ as a risk factor for duct-dependent pulmonary circulation after PBPV in PAIV [3].

- Composite scores and other indices

With the previously described limitations related to the use of Z-scores, both PV Z-scores [3,6] and interventricular septum diastolic thickness Z-scores [4] have been demonstrated to be other markers able to predict duct/shunt dependency of the pulmonary circulation in PA IVS/CPS. Giordano et al. [3] recently proposed a score using multiple echocardiographic measurements (e.g., TV < 8.8 mm, TV Z-score ≤ 2.12, TV/MV < 0.78, PV < 6.7 mm, PV Z-score ≤ 1.17, RVED area < 1.35 cm^2^, right atrial (RA) area > 2.45 cm^2^, % of PFO right-to-left shunt > 69.5%, moderate/severe TR, RV systolic pressure > 42.5 mmHg, tricuspid *E*/*E*′ ratio > 6), each of them assigning one point if reaching the cut-off value. A score ≥ 4 had a 100% sensitivity and 86% specificity in predicting the need for PDA stenting/shunt in 55 neonates with PA-IVS/CPS (Table 1) [3].

## 4. Severe Ebstein’s Anomaly

Despite the broad surgical literature on Ebstein’s’ anomaly [14,15,16,17], there are limited echocardiographic studies [18,19,20,21] aimed to evaluate the echocardiographic predictors for BVR in severe neonatal forms of Ebstein’s anomaly.

Celermajer’s index: In 1992 [18], Celermajer and colleagues published a famous score to grade Ebstein’s disease severity based on simple echocardiographic data. The score is calculated as the ratio of RA + atrialized RV to the functional RV + left atrium (LA) + LV. The study included 50 neonates with Ebstein’s anomaly evaluated in London from 1960 to 1990 [26]; however, only in 28 out of 50 cases were echocardiographic data available. Four neonates had a score of grade 1, who were all alive at a follow-up of 5 to 9 years; ten neonates had a score of grade 2, of which one died suddenly at 4 months; nine neonates had grade 3, of which five died (ranging from 4 months to 9 years of total life); and five neonates had grade 4, all of whom died within 18 months.

In 2013, Yu et al. [21] reviewed 59 cases of Ebstein’s anomaly from South Korea, reporting a mortality rate of 23.7%; however, neither the Carpentier’s classification (*p* = 0.175) nor the Celermajer’s index (*p* = 0.958) was significantly related to death. Instead, univariate analysis revealed that fetal distress (*p* = 0.002), prematurity (*p* = 0.036), low birth weight (*p* = 0.003), diameter of the ASD (*p* = 0.002), and pulmonary stenosis/atresia (*p* = 0.001) were related to mortality. On multivariate analysis, however, only fetal distress (*p* = 0.004) and pulmonary atresia/stenosis (*p* < 0.001) remained significant determinants of outcome [21]. Both studies [18,21] suffered from methodological limitations including being single-center in origin, retrospective in design, and including all neonates diagnosed without clear inclusion and exclusion criteria (all neonates with Ebstein’s anomaly regardless of the severity of the disease were included, including those with associated congenitally corrected transposition of the great arteries) [18,21]. Furthermore, surgical/interventional algorithms were not described but were certainly different from more recent ones. The study cohort in one paper [21] also had an uneven distribution of disease severity, with only eight (15.3%) and two neonates (3.8%) having grade II and grade IV of Celermajer’s index and/or type C and D of Carpentier’s classification, respectively [21].

In a retrospective surgical series from 2016 [17] on 12 neonates who were diagnosed with severe TR and pulmonary atresia related to Ebstein’s anomaly (*n* = 9) or isolated TV dysplasia (*n* = 3), six underwent a BVR and three survived [17]. A TR flow velocity > 3.0 m/s was an indicator of successful BVR [17]. A study evaluating the characteristics of Ebstein’s anomaly during fetal life revealed a series of independent predictors of mortality including gestational age < 32 weeks, TV Z-score, pulmonary regurgitation (all *p* < 0.001), and the presence of pericardial effusion (*p* = 0.04) [22].

## 5. Unbalanced AVSD

Unbalanced atrioventricular septal defect (UAVSD) with left dominance is usually defined when the atrioventricular valve index (AVVI) (left valve area/right valve area in subcostal view) is >0.6 [23,24,25,26]. Studies that assessed the ability of scores to predict successful BVR in UAVSD have excluded those with a left dominance [23], thus data for UAVSD with left dominance are extremely limited [25,26]. In 2015, Jegatheeswaran et al. [25] reviewed data from 58 patients with UAVSD (50 with right dominance and 8 with left dominance) treated at four different centers between 2000 and 2006. They observed that in patients with UAVSD with left dominance (e.g., AVVI of >0.6), surgical strategies varied and interim palliation (PA banding) and one-and-a-half ventricle repair strategies were preferred.

Later, Nathan et al. [26] reviewed 16 UAVSD cases (including 6 with left dominance) with prior single ventricle palliation who underwent conversion to BVR between 2003 and 2011 at the Children’s Hospital, Boston. Primary BVR or initial pulmonary artery banding or shunting with subsequent conversion to BVR was recommended for those with a mild hypoplasia of the ventricle (LV or RV volumes > 30 mL/m^2^) or AVVI (0.19 < AVVI < 0.39 or 0.61 < AVVI < 0.80. 60% to 80% overriding,) and apex-forming ventricles [26]. In contrast, in UAVSD patients with the same AVV characteristics but moderate ventricular hypoplasia (RV/LV 15 to 30 mL/m^2^) and near apex-forming ventricles, single ventricle palliation and ventricular recruitment with subsequent staged biventricular conversion was advised, whereas univentricular palliation was advised only in UAVSD with severe ventricular hypoplasia (LV/RV < 15 mL/m^2^, 0.19 < AVVI > 0.81, 80% overriding) [26].

## 6. Discussion

Although echocardiography is often the primary and sometimes only imaging modality for pre-surgical/interventional diagnosis in complex neonatal CHDs characterized by borderline RV, the echocardiographic indicators available for risk prediction in this cardiac lesion remain limited.

All the literature data reviewed herein showed significant methodological limitations, including a single-center design [3,4,5,6,7,8,9,10,11,12,13,18,21,25,26], retrospective design [3,4,5,6,7,8,9,10,11,13,18,21,25,26], poor definition of inclusion/exclusion criteria [3,4,5,6,7,8,9,10,11,12,13,18,21,25,26], the lack of interventional algorithms, and a clearly limited [4,5,7,9,10] or relatively limited sample size [2,6,8,11]. Furthermore, studies [7,8,11,12,13,19] reviewing the surgeries/interventions of two or more decades ago may have little clinical relevance now.

A series of echocardiographic parameters have been proposed for BVR risk assessment in patients suffering from PAIVS/CPS [1,2,3,4,5,6,7,8,9,10,11,12,13]. However, it is difficult to establish which of the parameters proposed should be recommended for use in daily practice. TV/MV [4,5,13] and RV/LV [9] diameter ratio can be readily acquired and very reproducible indices that show a good accuracy for the prediction of successful BVR or the need for pulmonary blood flow augmentation after PBVP. However, data are very limited, with three studies enrolling less than 30 cases [3,5,9]. In larger studies [1,2,3] (up to 99 investigated cases), the RVED area has been demonstrated as an accurate predictor of BVR [1,2] or the need for pulmonary blood flow augmentation [3]. The RVED area, however, may be less reproducible and very dependent on load conditions, thus discordant cut-off values have been reported by different studies [1,3]. Similarly, it has been demonstrated that a higher degree of TR is favorable for BVR [1,2]. However, estimation of TR in the neonatal age is highly subjective and very dependent on load/pressure conditions, the pulmonary vascular bed, and ventilatory support. Data that focused on TV Z-scores [4,6,8,13] may have significant limitations, since the nomograms that were employed [28,29,30,31] are different from those currently employed [27,32]; thus, the cut-off values are poorly applicable in daily practice.

The use of composite scores with multiple markers, as recently proposed, seems to be a reasonable approach for future studies [3]. There is a need for large prospective studies with clear endpoints and clear inclusion/exclusion criteria. Furthermore, the timing of echocardiographic examination (before and/or after PBPV), measurement, and quantification techniques also needs to be standardized. Lastly, attention to confounders such as the strategies for pulmonary blood flow augmentation (BTM of duct stenting), criteria for re-intervention, and definition of a successful BVR need to be specified.

In summary, there is a shortage of echocardiographic studies clarifying prognostic indicators in other forms of borderline RV including Ebstein’s anomaly (and other forms of tricuspid valve dysplasia) [18,19,20,21] and UAVSD [23,24,25,26]. Although widely used, the prognostic value of the Celermajer index [17] for BVR in severe neonatal Ebstein’s anomaly has been poorly validated in clinical studies. The score may also present limitations due to the dependency on cardiac load and pressure [33,34] in chamber area. The level of agreement between the Celermajer score determined by echocardiography and that obtained through MRI is only moderate at best (kappa coefficient = 0.39, *p* = 0.002). Typically, echocardiography tends to overestimate the severity of Ebstein’s anomaly, although underestimations can rarely occur [18].

The available literature describing UAVSD is extremely limited [25,26], since the studies aiming to investigate echocardiographic indicators for BVR in this specific population excluded or do not analyze UAVSD with left dominance. Additionally, data on rare defects, such as isolated RV hypoplasia [36], that may present with cyanosis in the neonatal age are also very limited [36].

## 7. Conclusions

Our review summarizes the strengths and limitations of the current echocardiographic indices to predict successful BVR and the need for reintervention/pulmonary blood flow stabilization in complex neonatal CHD with borderline RV, with a specific focus on PAIVS and CPS. Examining the echocardiographic evaluation of disease severity is crucial for complex CHD’s encountered in daily practice and should serve as a guide for further research that would appear to be necessary based on the observations from this review.

## Figures and Tables

**Table 1:** Major studies evaluating echocardiographic parameters for risk prediction in PA IVS/CPS.

	Population	End Point	Echo Predictors
Cho MJ 2013 South Corea [4]	9 PA IVS, 13 CPS Undergoing PBPV −10 need aPBF Age 4.89 ± 2.47 days Weight 2.98 ± 0.6 kg −10 do not need aPBF Age 10.10 ± 10.63 days Weight 3.3 ± 0.3 kg	Need of PDA stenting or shunt	TV z-score ≤ −0.74 (AUC 0.8; specificity 90%, sensitivity 77.8%, *p* = 0.0189) TV/MV ≤ 0.9, (AUC 0.939; specificity 90%, sensitivity 88.89%; *p* < 0.0001) z-score IVSD ≥ 2.37 (AUC 0.804; specificity 75%, sensitivity 85.71%; *p* = 0.0222)
Chen RHS 2018 Hong Kong [5]	36 PA IVS Undergoing PBPV −26 BVR Age 5 (1–83) days Weight 3.21 ± 0.55 kg −5 no BVR Age 4 (2–51) days Weight 2.8 ± 0.31	Good BVR outcome	TV/MV > 0.79 AUC 0.858 specificity 100%, sensitivity 70%, PPV 100%, NPV 50%)
Yucel IK, 2016 Turkey [6]	56 CPS Undergoing PBPV Age 7 (2–28) days. Weight 3.1 (1.6–4.5) kg NR 34 do not need aPBF Weight 3.07 ± 0.4 kg NR 21 need aPBF Weight 3.17 ± 0.4 kg	Need of PDA stenting or shunt Need of re-intervention	TV Z score < −1.93 (AUC = 0.696, specificity 84.4%, sensitivity 63.2%, *p* = 0.022) PV Z score < −1.69 (AUC = 0.72, specificity 64.7%, sensitivity 74%, *p* = 0.008) Bipartite RV (odds ratio 9.6).
Alwi M 2005 Malaysia [8]	53 PA IVS Undergoing PBPV −10 need aPBF Age 8 days (3 days–7 months) Weight 3.1 (2.4–6.8) kg −37 do not need aPBF Age 7 days (1 day–8 years) Weight 3.3 (2–18) kg	Need of PDA stenting or shunt	Lower TV z score TV Z-score −1.1 ± 1.47 Need of aPBF TV Z-score −0.58 ± 1.18 No need of aPBF
Drighil A, 2009, USA [9]	26 PA IVS Undergoing PBPV Age 6 (1–49) days −13 successful BVR Age 14.5 ± 9.0 days Weight 3.3 ± 0.6 kg −13 unsuccessful BVR Age 17.6 ± 15.3 days Weight 3.1 ± 0.6 kg	PBPV success	(1) RV/LV diameter > 0.76 predicts a 92.3% success rate. (2) RV/LV diameter ≤ 0.70 + RV/LV length ≤ 0.76 predicts 100% failure (3) RV/LV diameter ≤ 0.76 and RV/LV length > 0.70, 75% success rate
Schwartz MC, 2006, USA [10]	23 PA IVS undergoing RFV Age 2 (−16) days Weight 3.1 (2.1–4.1) kg	Need of re-intervention	Lower post-procedural PV PG (*p* = 0.05) TV z-score < −0.7 (*p* = 0.08)
Cleuziou J, 2010, Germany [11]	86 PA IVS 55 underwent PVVP (16 plus shunt) 26 underwent shunt. BVR in 56 Age 2.2 ± 4.8 years Shunt palliation in 13 UV in 17	Predictors of BVR Predictors of mortality	RV decompression ± shunt (*p* < 0.001), Tripartite RV (*p* < 0.001) No coronary fistulae (*p* < 0.001). At univariate analysis TV z score < 5, unipartite RV, coronary fistula, Ebstein’s, RV dependent coronary circulation, connection of the fistula with LCA and RCA
Maskatia SA, 2018, USA [1]	81 PA IVS Undergoing PBPV Age 3 (2–4) days	BVR, RV growth	Baseline RV area ≥ 0.6 cm^2^/m^2^ (Sensitivity 93%, specificity 80%, AUC 0.88, odds ratio 50.4) Follow-up RV area ≥ 0.8 cm^2^/m^2^ sensitivity 100% specificity 100%, AUC 0.96, odds ratio 67) More than moderate TR
Minich LL,2000, USA [13]	23 successful surgical BVR ° Age 11–20 days Weight 3.5 ± 0.6 kg 13 unsuccessful BVR Weight 2.9 ± 0.5 kg	BVR	Greater pre-op weight TV z score > −3, TV/MV > 0.5
Petit CJ, 2017, USA [2]	99 PA IVS undergoing PBPV Age 3 (2–5) days Weight 3.3 (2.7–3.7) kg	Primary: Reintervention post-RV decompression. Secondary: BVR	Virtual atresia (HR, 0.51; 95% CI, 0.28–091; *p* = 0.027), Smaller RV length (HR, 0.94; 95% CI, 0.89–0.99; *p* = 0.027), ≤Mild TR (HR, 3.58; 95% CI, 2.04–6.30; *p* < 0.001). ≤Mild TR (OR, 18.6; 95% CI, 5.3–5.2; *p* < 0.001) Lower RV area (OR, 0.81; 95% CI, 0.72–0.91; *p* < 0.001).
Giordano M, 2022, Italy [3]	55 PA IVS or CPS Age NR Weight 2.9 ± 5 kg 27 need aPBF Weight 3.0 ± 0.4 kg 28 do not need aPBF Weight 2.9 ± 0.6 KG	Need of PDA stenting or shunt	Composite score including TV < 8.8 mm, TV z-score ← 2.12, TV/MV <0.78, PV < 6.7 mm, PV z-score ←1.17, RVED area < 1.35 cm^2^, RA area > 2.45 cm^2^, % of PFO right-to-left shunt > 69.5%, moderate/severe TR, RV systolic pressure > 42.5 mmHg, tricuspid E/E′ ratio > 6.6 A score ≥ 4 sensitivity 100% and specificity 86%

Legend to Table: aPBF = adjunctive pulmonary blood flow, AUC = area under the ROC curve, BVR = biventricular repair; CI = confidence interval, CPS = critical pulmonary stenosis, HR = hazard ratio, IVSD = end-diastolic interventricular septal thickness; LCA = left coronary artery; LV = left ventricle; MV = mitral valve; NR = number; OR = odds ratio; PDA = patent arterial duct, PFO = patent foramen ovale: PV = pulmonary valve, PG = pressure gradient, PBPV = percutaneous balloon pulmonary valvuloplasty, RCA = right coronary artery; RV = right ventricle; RVED = right ventricle end-diastolic; TR = tricuspid regurgitation; TV = tricuspid valve; ° closed trans-ventricular valvulotomy with and central shunt.

## Data Availability

The data presented in this study are available on request from the corresponding author.

## Supplementary Materials

The following supporting information can be downloaded at: https://www.mdpi.com/article/10.3390/jcm12144599/s1. Table S1: Inclusion/exclusion criteria in major studies evaluating echocardiographic parameters for risk prediction in PA IVS/CPS. References [1,2,3,4,5,6,8,9,10,11,13] are cited in Supplementary Materials.
