# Supplementary material for: Echocardiographic Parameters for Risk Prediction in Borderline Right Ventricle: Review with Special Emphasis on Pulmonary Atresia with Intact Ventricular Septum and Critical Pulmonary Stenosis
Source: J Clin Med. 2023 Jul 10;12(14):4599. doi: 10.3390/jcm12144599 (PMC10380858; doi:10.3390/jcm12144599)
Supplement: Supplementary file 1 [file jcm-12-04599-s001.zip › jcm-2402741-supplementary.pdf]

Table S1: Inclusion/exclusion criteria in major studies evaluating echocardiographic parameters for risk prediction in PA IVS/CPS

	**Inclusion criteria**	**Exclusion criteria**
Cho MJ 2013 South Korea [4]	PA IVS; CPS	Failure to perforate the PV
Chen RHS 2018 Hong Kong [5]	PA IVS who attempt CBPV	Patients who received a previous shunt Unipartite RV, RV dependent coronary circulation, muscular type atresia, significant TV anomalies (such as Ebstein's anomaly)
Yucel IK, 2016 Turkey [6]	CPS	Non-critical PS
Alwi M,2005, Malaysia [8]	PA IV RFV	None
Drighil A 2009, USA [9]	PA IVS undergoing CBPV	Diminutive RV, TV z score <3, absence or RV infundibulum
Schwartz MC,2006, USA [10]	PA IVS undergoing RFV	Ebstein's anomaly
Cleuziou J, 2010 Germany [11]	PA IVS	None
Maskatia SA, 2018, USA [1]	PA IVS underwent decompression at < 30 days	CPS, Ebstein's anomaly
Minich LL 2000 USA [13]	PA IVS<br>Attempting surgical opening RVOT	NR
Petit CJ 2017 [2]	PA IVS<br>Candidates for RV decompression (either surgical or percutaneous, underwent intervention within 30 days)	CPS, Ebstein's anomaly RV decompression after neonatal age
Giordano M 2022 Italy [3]	PA IVS, CPS	Severe RV hypoplasia, RV dependent coronary circulation

Legend to Table: CPS= critical pulmonary stenosis, CBPV= catheter balloon valvuloplasty; PPV=positive predictive value, NPV=negative predictive value, NR=not reported, SPS=severe pulmonary stenosis, RV=right ventricle; RFV=radio-frequency perforation and valvuloplasty, RVOT=right outflow tract.
